# Supplementary material for: Senescent tumor cells lead the collective invasion in thyroid cancer
Source: Nat Commun. 2017 May 10;8:15208. doi: 10.1038/ncomms15208 (PMC5436223; doi:10.1038/ncomms15208)
Supplement: Supplementary Information — Supplementary Figures and Supplementary Tables [file ncomms15208-s1.pdf]

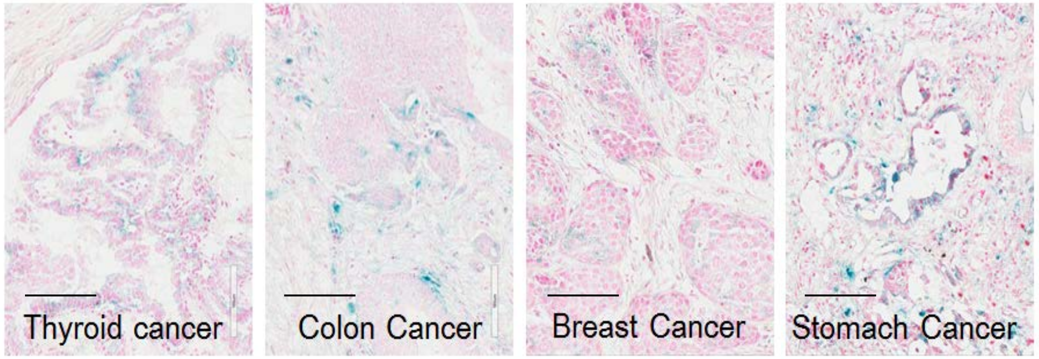

Supplementary Figure 1. SA-β-Gal positive senescent cells in various cancer tissues. Representative frozen sections of breast, thyroid, colon and stomach cancer were stained with SA-β-Gal and nuclear fast red (NFR) as counterstaining. Bar indicates 100 μm

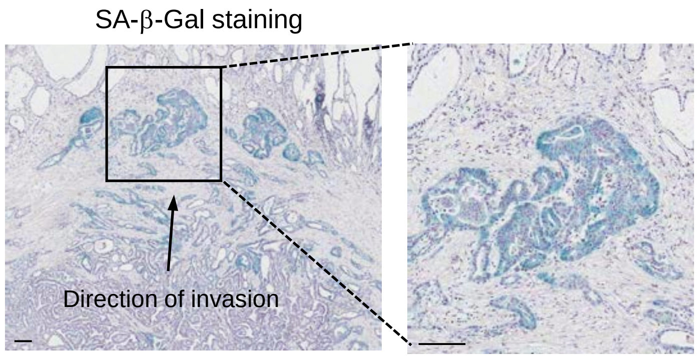

Supplementary Figure 2. SA-β-Gal positive senescent cells at the invasive border of BRAFV600E-expressing PTC. PTC frozen sections were stained with SA-β-Gal and hematoxylin as counterstaining. Bar indicates 100 μm.

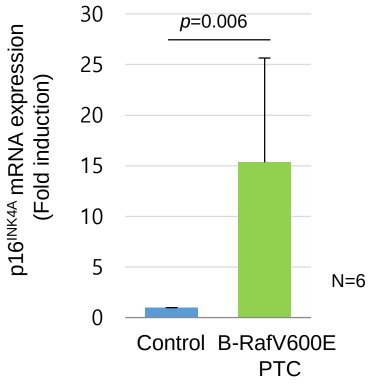

Supplementary Figure 3. *p16*^{INK4A} mRNA expression was analyzed in BRAFV600E-expressing PTC and adjacent normal tissues by real-time PCR and presented as a bar graph (n=6). The values indicate the relative values of PTC compared with the normal follicle. The *p* values were calculated by Student's *t*-test. Error bars, s.d.

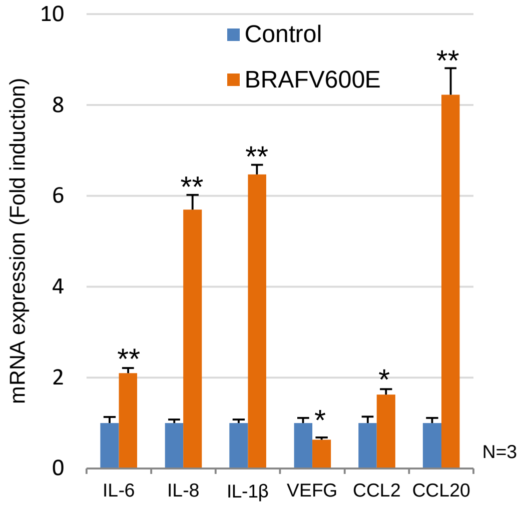

Supplementary Figure 4. SASP expression was analyzed in normal and *BRAFV600E*-induced senescent thyrocytes by real-time PCR (n=3). Normal thyrocytes were infected with control or *BRAFV600E* lentivirus for 10 days and then *IL-6*, *IL-8*, and *CCLs* expression were analyzed. The *p* values (*; $p<0.001$, **; $p<0.0001$) were calculated by Student's *t*-test. Error bars, s.d.

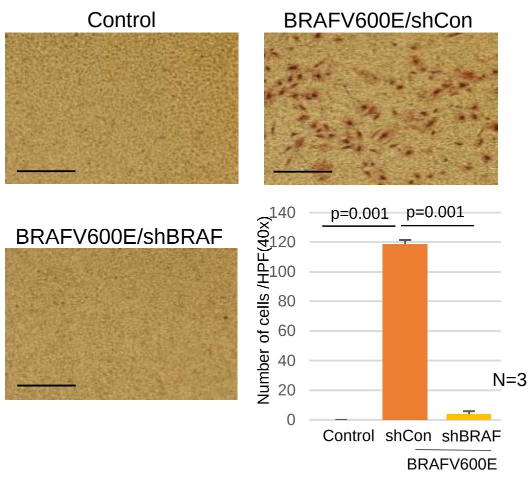

Supplementary Figure 5. Senescent cells have a high invasive ability. Normal thyrocytes were infected with control, *BRAFV600E*, or *BRAFV600E/shBRAF* lentivirus for 10 days, and then invasion assay were performed. Control, *BRAFV600E*, or *BRAFV600E/shBRAF* lentivirus infected thyrocytes were seeded on transwells. After 24 hr, cells which have invaded the lower surface of the filters were counted. The number of migrated cells was counted in the 40x magnification field. Independent experiments were performed and presented in the bar graph (n=3). Bar indicates 100 μm. The *p* values were calculated by Student's *t*-test. Error bars, s.d.

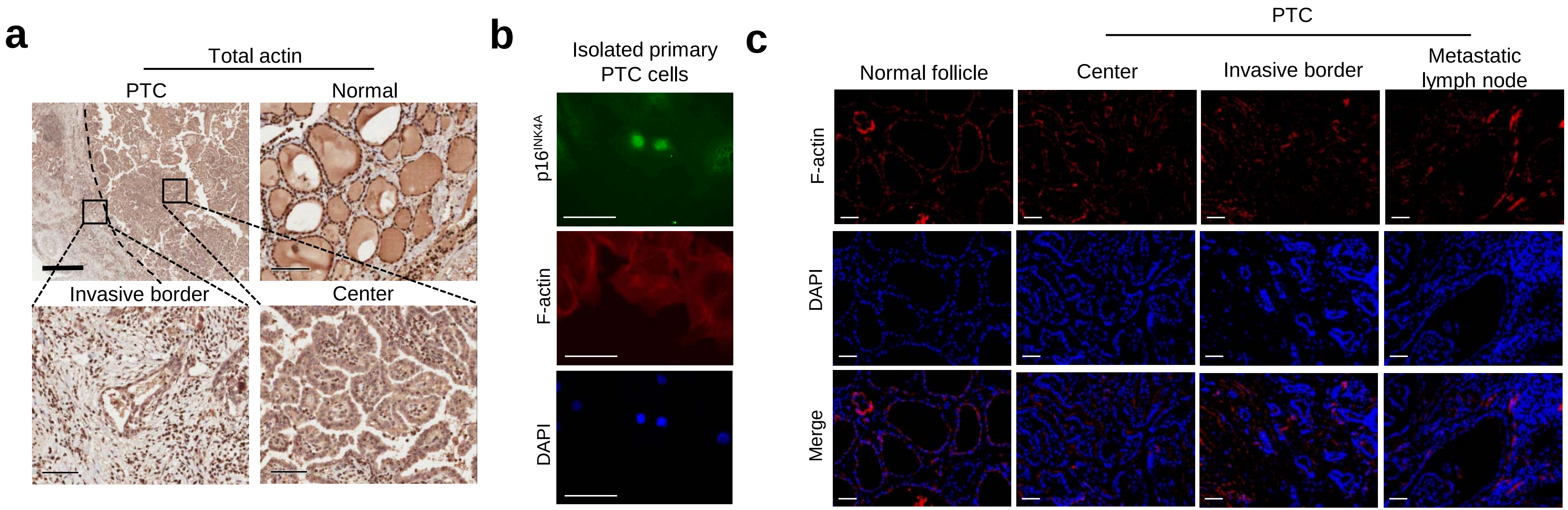

Supplementary Figure 6. Analysis of actin polarization in invasive border of PTC. (a) Immunohistochemical analysis of actin expression in BRAFV600E-expressing PTC. Normal follicles, center, and invasive regions of cancer were analyzed. (b) F-actin expression in isolated PTC tumor cells. Tumor cells were isolated from BRAFV600E-expressing PTC and stained with p16^{INK4A} and phalloidin. (c) F-actin expression in normal follicle, cancer center and invasive region. BRAFV600E-expressing PTC slides were stained with phalloidin for 1 hr. Images of a normal follicle, center, and invasive area of cancer were acquired by fluorescence microscope. Bar in (a): left upper bar indicates 1mm and others indicate 100 µm. Bar in (b) and (c) indicates 50 µm.

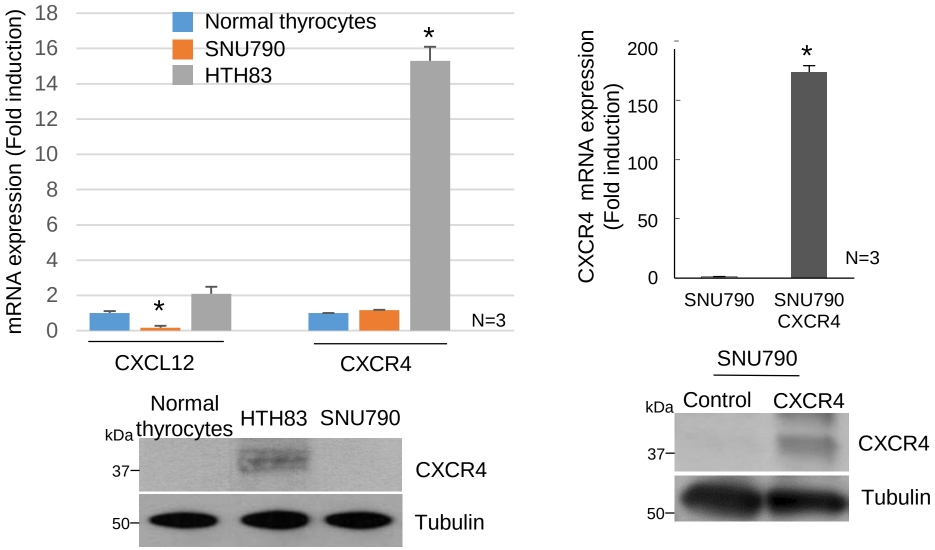

Supplementary Figure 7. CXCR4 and CXCL12 expression in normal thyrocytes, SNU790 and HTH83 cells. *CXCR4* and *CXCL12* mRNA expression was analyzed in normal thyrocytes, SNU790 cells, and HTH83 cells by real-time PCR (n=3, left upper panel) and western blotting (left lower panel), respectively. SNU790 cells were infected with control or *CXCR4* lentivirus and then selected with puromycin for 3 days. *CXCR4* expression was then analyzed by real-time PCR (n=3, right upper panel) and western blotting (right lower panel), respectively. The *p* values (*; $p<0.001$) were calculated by Student's *t*-test. Error bars, s.d.

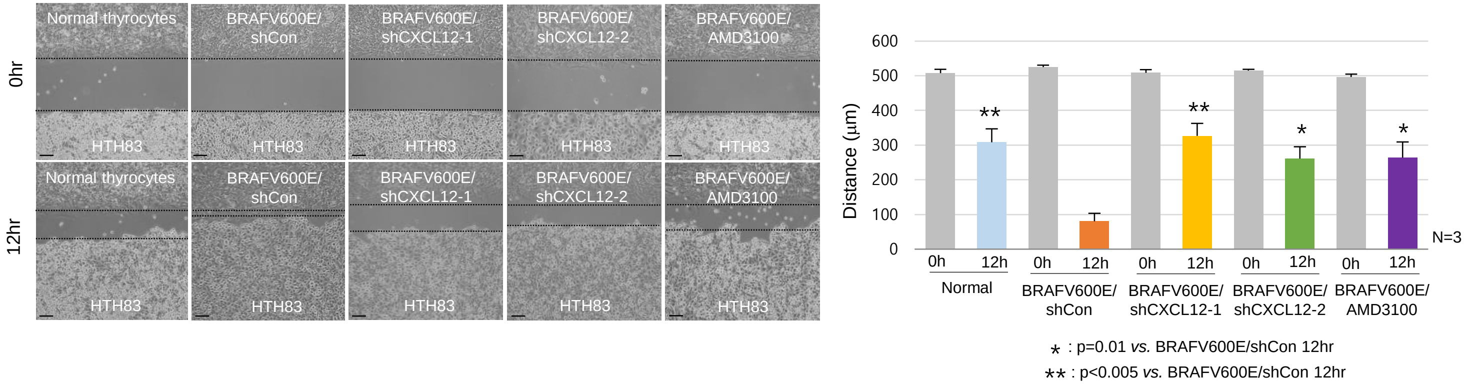

Supplementary Figure 8. *In vitro* cell migration assay. Normal/HTH83, *BRAFV600E*/HTH83, and *BRAFV600E-shCXCL12*/HTH83 cells were seeded in 2 well of 3.5 cm dishes and removed the well barrier. After 12 hr, cell migration was measured. One set of *BRAFV600E*/HTH83 was treated with 1μM of AMD3100 to inhibit CXCR4 signaling. The bar graph indicates the average of independent measurements (n=3). The *p* values were calculated by Student's *t*-test. Bar indicates 100 μm. Error bars, s.d.

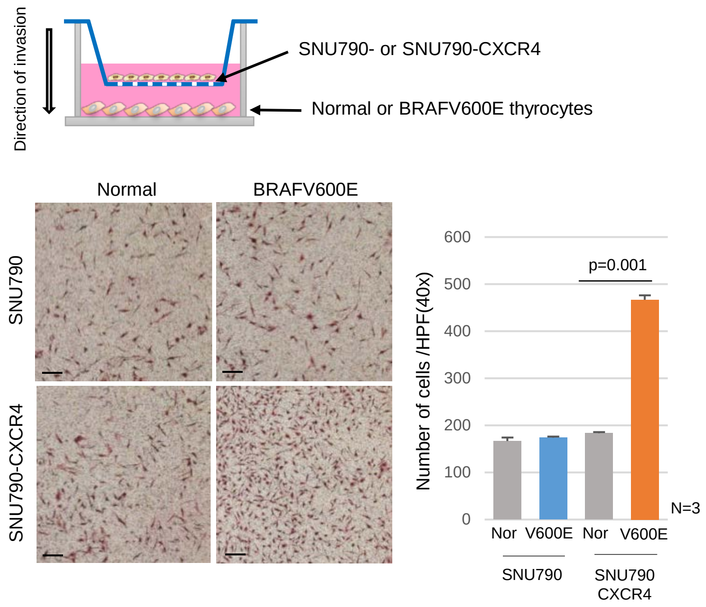

Supplementary Figure 9. Transwell assay. SNU790 or SNU790-*CXCR4* cells suspended in medium were seeded on transwells. Control or *BRAFV600E* lentivirus infected thyrocytes were seeded at the bottom. After 24 hr, cells which have invaded the lower surface of the filters were counted. The number of migrated cells was counted in the 40x magnification field. Independent experiments were performed and presented in a bar graph (n=3, right panel). The *p* values were calculated by Student's *t*-test. Bar indicates 100 μm. Error bars, s.d.

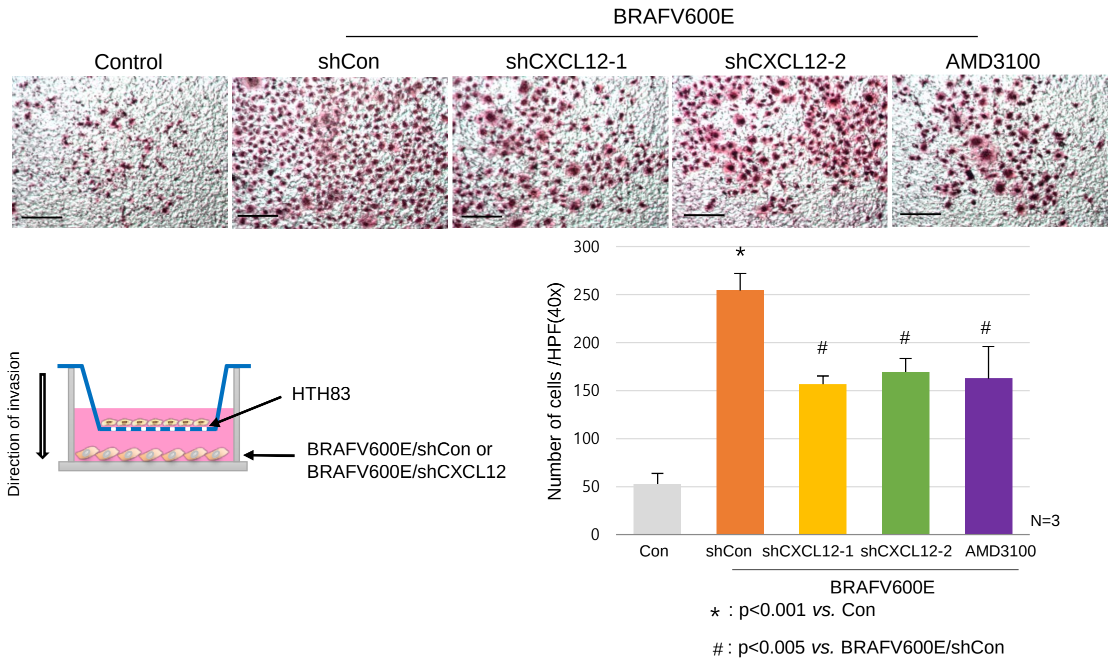

Supplementary Figure 10. Transwell assay. HTH83 cells suspended in medium were seeded on transwells. Control, *BRAFV600E*, *BRAFV600E/shCXCL12*, or *BRAFV600E*/AMD3100 treated cells were seeded at the bottom. After 12 hr, cells which have invaded the lower surface of the filters were counted. The number of migrated cells was counted in the 40x magnification field. Three independent experiments were performed and presented in a bar graph (n=3, right panel). The *p* values were calculated by Student's *t*-test. Bar indicates 100 μm. Error bars, s.d.

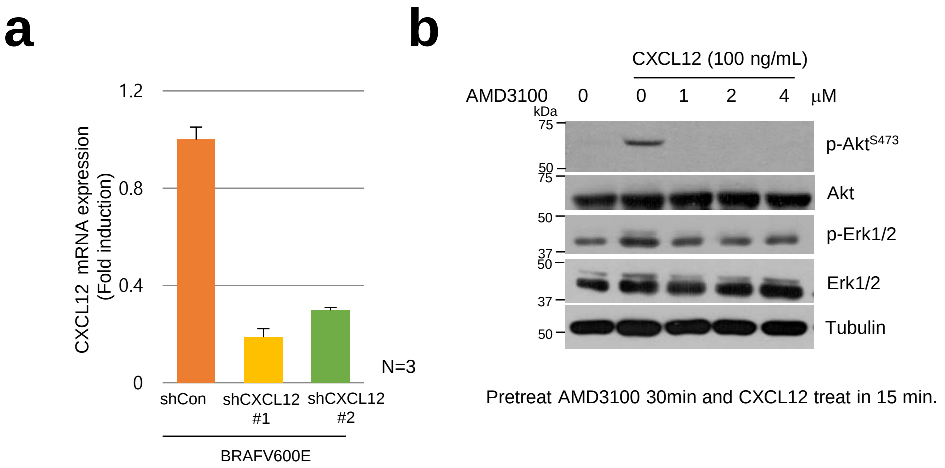

Supplementary Figure 11. (a) *CXCL12* mRNA expression in *CXCL12* knockdown thyrocytes. *BRAFV600E* induced senescent thyrocytes were infected with shCon or two kinds of *shCXCL12* lentivirus and then analyzed *CXCL12* mRNA expression by real-time PCR (n=3). (b) HTH83 cells were treated with recombinant CXCL12 protein (100 ng/ml) for 15 min and then analyzed p-Erk1/2 and p-Akt^{S473} by western blotting. In the case of inhibition study, HTH83 cells were pretreated with AMD3100 for 15 min with indicated concentration (0 to 4 μM). Error bars, s.d.

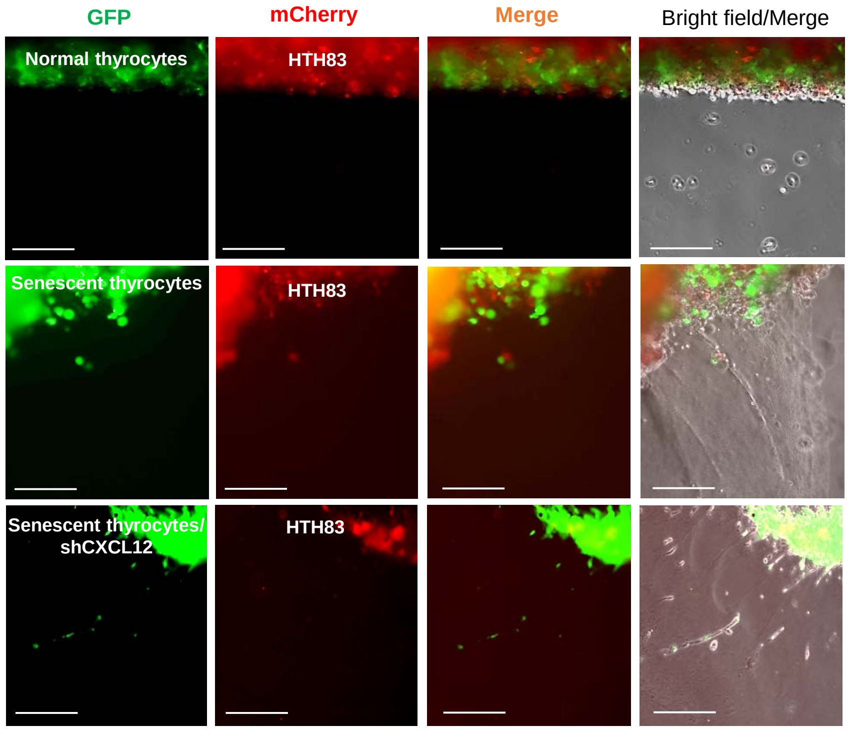

Supplementary Figure 12. Three-dimensional invasion assay. mCherry lentivirus-infected HTH83 cells were co-cultured with GFP lentivirus-infected normal (upper panel), *BRAFV600E* (middle panel), or *BRAFV600E/shCXCL12* thyrocytes (lower panel) on the top of collagen I containing matrigel for 48 hr. Bars indicate 50 μm.

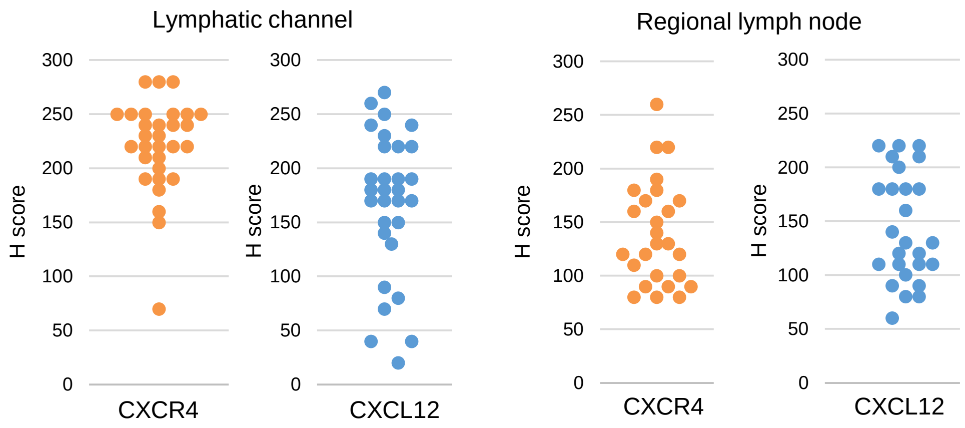

Supplementary Figure 13. Analysis of CXCL12/CXCR4 immunohistochemical staining in metastatic foci of regional lymph nodes and in tumor emboli of lymphovascular spaces in *BRAFV600E*-expressing PTC. Analysis of CXCL12 and CXCR4 immunohistochemical staining was assessed by H score in 26 metastatic foci of regional lymph nodes and 30 tumor emboli in lymphovascular spaces of BRAFV600E-expressing PTC.

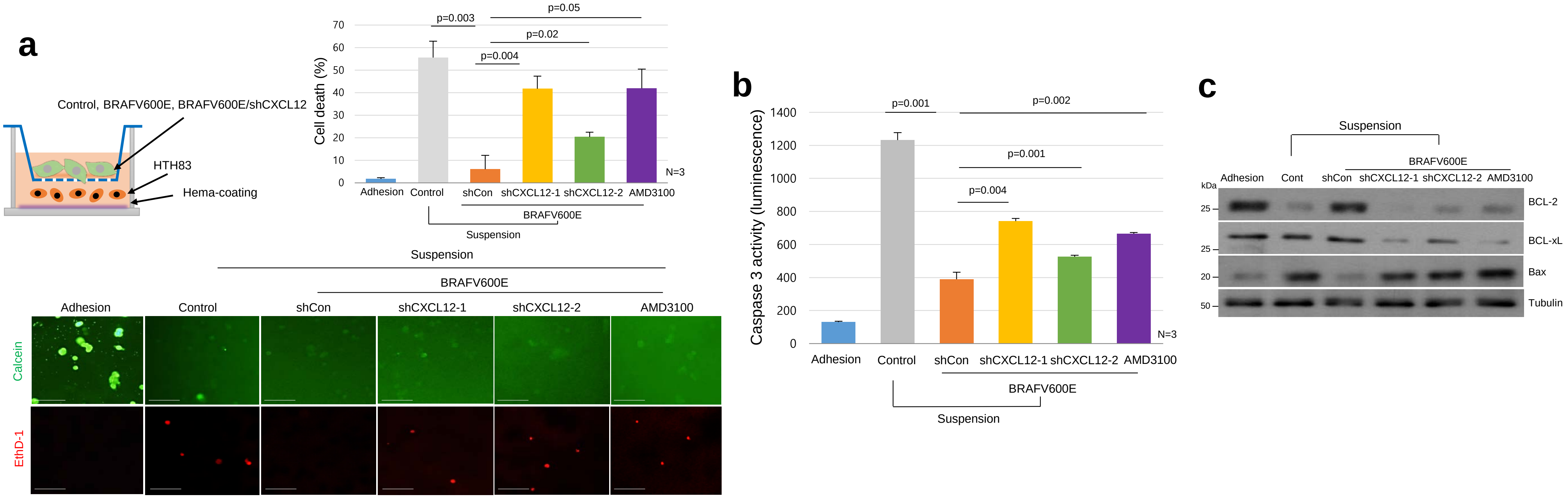

Supplementary Figure 14. Anoikis inhibitory function of senescent cells. Control, *BRAFV600E*/shCon, *BRAFV600E/shCXCL12*, and *BRAFV600E*/AMD3100 treated cells were co-cultured with HTH83 cells in HEMA-coated plate for 12 hr and cell death was determined by Calcein AM and EthD-1 staining (a), caspase activity (b) and apoptosis related proteins expression (c). Independent experiments were performed and presented in a bar graph (n=3). The *p* values were calculated by Student's *t*-test. Bar indicates 50 μm. Error bars, s.d.

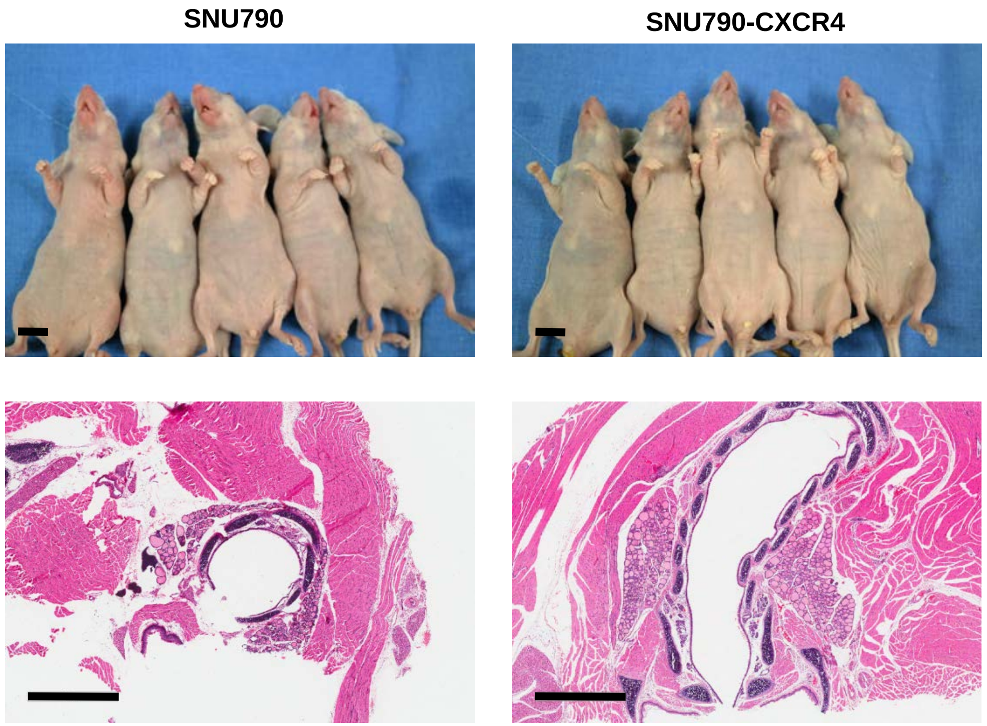

Supplementary Figure 15. Thyroid tumors developed in female nude mice. 1×10^6 cells (SNU790 or SNU790-*CXCR4* cells) were transplanted into the thyroid gland of 9-week-old female nude mice. The mice were euthanized after 3 weeks and the incidence of tumor development were analyzed by HE staining. Bar in upper panel indicates 10 mm. Bar in lower panel indicates 1 mm.

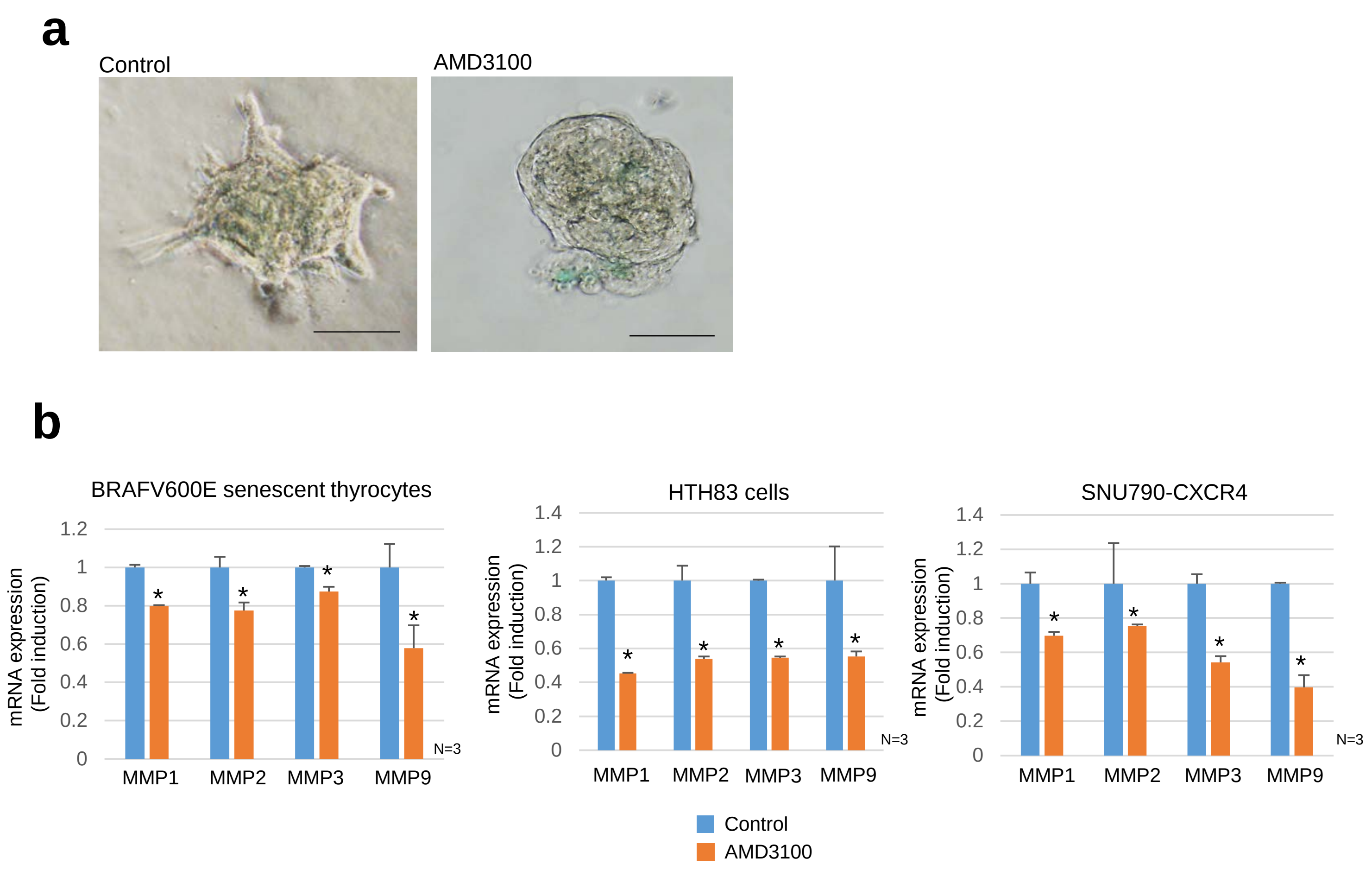

Supplementary Figure 16. Inhibition of *CXCR4* decreased cancer cells invasion in organoid culture. (a) PTC were digested with collagenase to obtain small fragments of cancer tissues. The fragments were cultured in collagen I containing matrigel for 96 hr with/without 1μM AMD3100 and then SA-β-Gal staining was performed. (b) *MMPs* expression was analyzed in *BRAFV600E*-induced senescent thyrocytes, HTH83 and SNU790-*CXCR4* cells. The cells were treated with/without 1μM of AMD3100 for 48 hr and then analyzed *MMPs* mRNA expression by real-time PCR. Independent experiments were performed and presented as a bar graph (n=3). The *p* values (*; $p<0.01$) were calculated by Student's *t*-test. Error bars, s.d.

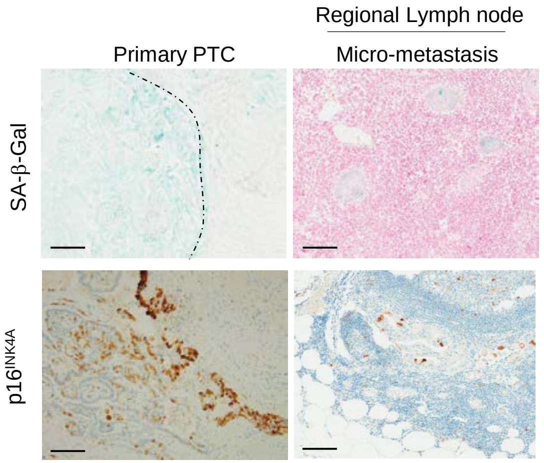

Supplementary Figure 17. SA-β-Gal staining in primary PTC and micrometastatic foic of lymph node. Frozen section of BRAFV600E-expressing PTC regional lymph node was stained with SA-β-Gal. Bar indicates 100 μm.

Fig.3e

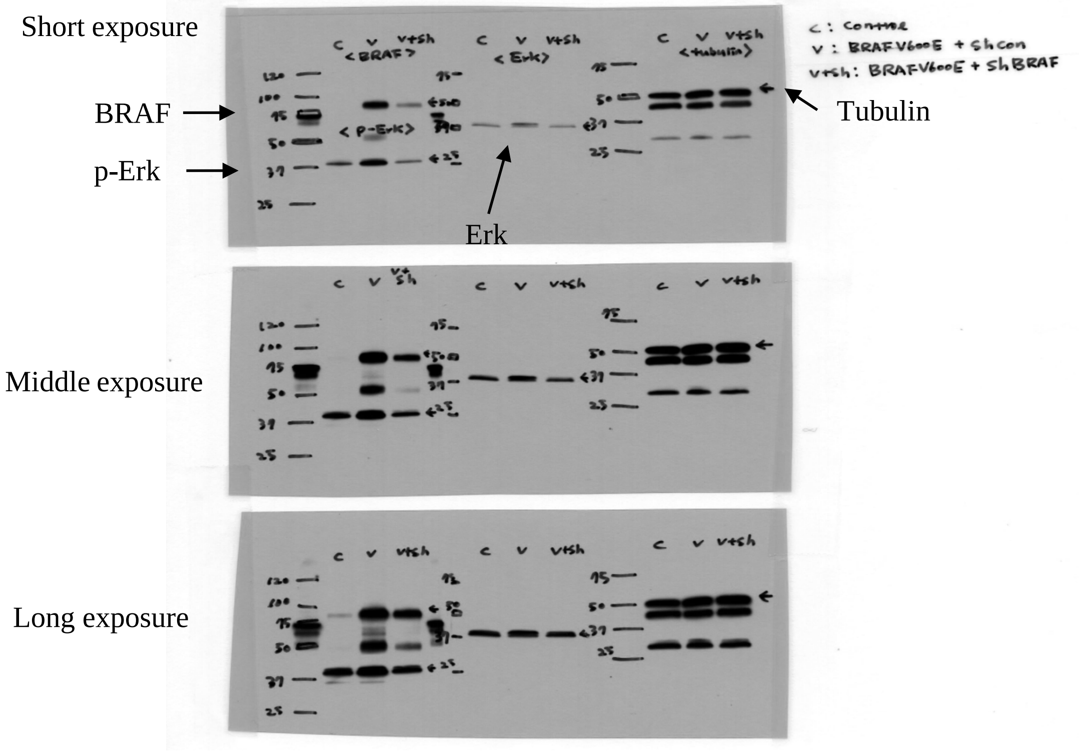

Fig.3g

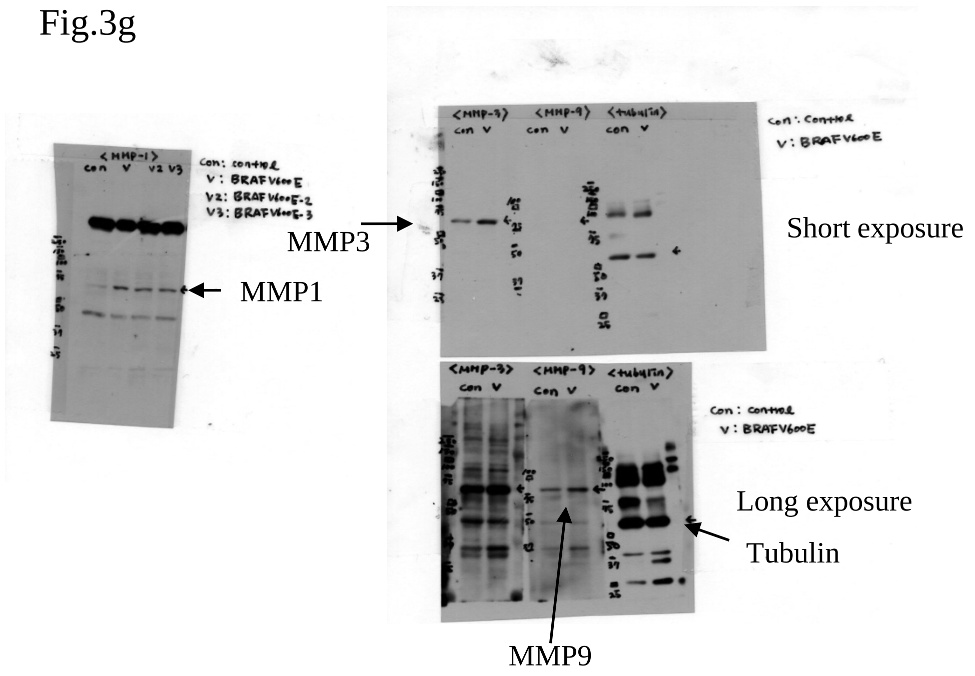

Supplementary Figure 18. Uncropped scans of the Western blots shown in the indicated figures.

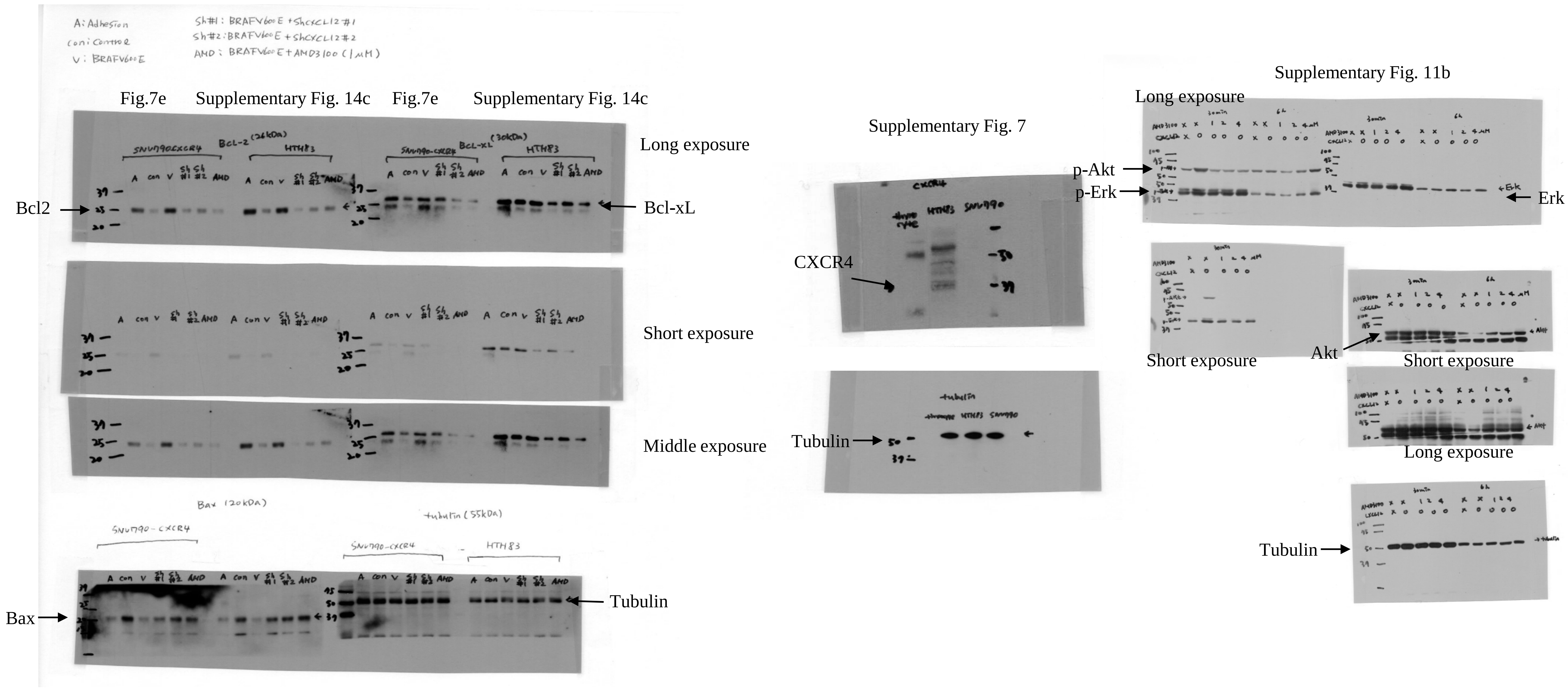

Supplementary Figure 19. Uncropped scans of the Western blots shown in the indicated figures.

Genes	Number of case	Average of fold induction	STDEV	P value
**MMP1**	25	11.2	12.8	0.0002
**MMP3**	13	5.3	7.9	0.038
**MMP9**	13	12.7	14.3	0.005
**IL-6**	13	9.8	11.7	0.008
**IL-8**	13	6.9	10.6	0.033
**CCL20**	13	15.6	18.4	0.005

Supplementary Table 1. SASP expression in *BRAFV600E*-expressing PTC. SASP expression was analyzed in 13 cases of *BRAFV600E*-expressing PTC and adjacent normal tissues by real-time PCR and presented the results as a table. The values indicate the relative value compared to that of the normal follicle. In the case of *MMP1*, 25 cases were analyzed.

Case No	Total LN	Metastatic LN	$p16^{INK4A}$ Positive LN (%)
FFPE case 1	5	4	4 (100)
FFPE case 2	6	4	4 (100)
FFPE case 3	2	2	2 (100)
FFPE case 4	10	2	2 (100)
FFPE case 5	9	2	2 (100)
FFPE case 6	11	5	5 (100)
FFPE case 7	7	3	3 (100)
FFPE case 8	5	5	5 (100)
FFPE case 9	1	1	1 (100)
FFPE case10	1	1	1 (100)
Total	57	29	29 (100)

Case No	Total LN	Metastatic LN	SA-β-Gal positive LN (%)
Frozen case 1	1	1	1 (100)
Frozen case 2	2	1	1 (100)
Frozen case 3	3	2	2 (100)
Frozen case 4	8	1	1 (100)
Frozen case 5	3	1	1 (100)
Frozen case 6	2	2	2 (100)
Frozen case 7	1	1	1 (100)
Frozen case 8	2	1	1 (100)
Frozen case 9	2	2	2 (100)
Frozen case 10	2	2	2 (100)
Total	26	14	14 (100)

Total LN : Number of total lymph node examined
Metastatic LN: Number of metastatic lymph node among total lymph nodes
$p16^{INK4A}$ Positive LN/SA-β-Gal positive LN : Number of metastatic lymph node which include $p16^{INK4A}$ Positive/ or SA-β-Gal positive tumor cells

Supplementary Table 2. Senescent tumor cells in regional lymph nodes. To examine senescent tumor cells in metastatic regional lymph nodes, SA-β-Gal (right panel), and $p16^{INK4A}$ (left table) staining were performed in ten cases of PTC. Numbers of lymph node including SA-β-Gal positive or $p16^{INK4A}$ positive tumor cells were presented as a table.

Gene	Patient 1		Patient 2		Patient 3	
	Center	Invasive	Center	Invasive	Center	Invasive
CXCL1	0	0.0393686	0.235351	0.0944145	0.165317	0.456146
CXCL2	1.12349	1.90469	1.8666	14.0182	0.692885	6.69048
CXCL3	0.337034	0.160092	0.0478391	0.671839	0.16803	0.502202
CXCL5	0	0.1379	0.0205701	0	0.0481255	0.0331918
CXCL6	0	0.0793826	0	0	0.0369755	0
CXCL9	3.45349	2.34161	1.3872	3.59152	13.9088	33.0471
CXCL10	1.63821	1.05342	1.169	2.70562	8.79027	16.01
CXCL11	0.194008	0.110122	0.131509	0.362423	2.30846	2.36169
CXCL12	11.6229	16.9109	11.6017	31.2645	62.7624	85.164
CXCL13	0.160498	0.266656	0.455267	0.639269	42.6923	104.548
CXCL16	38.3676	27.1628	22.4343	23.1729	38.4028	21.0784

Gene	Patient 1		Patient 2		Patient 3	
	Center	Invasive	Center	Invasive	Center	Invasive
CXCR1	0.0722612	0.0510885	0.0812864	0.101743	0.0475437	0.0819763
CXCR2	0.134385	0.142457	0.0944337	0.132371	0.198834	0.243794
CXCR3	1.45142	1.36052	1.35901	1.30202	4.6196	5.40081
CXCR4	5.58792	7.64973	20.7405	14.2627	33.1371	81.4413
CXCR5	0.0205837	0.0727657	0.0173436	0.0173628	4.23036	6.97455
CXCR6	0.1873	0.46427	0.343091	0.766674	4.53911	6.02697
CXCR7	2.77063	5.47468	12.0208	13.2121	6.50401	12.2966

Gene	Patient 1		Patient 2		Patient 3	
	Center	Invasive	Center	Invasive	Center	Invasive
CCL1	0	0	0	0	0	0
CCL2	21.0781	11.6244	35.4983	54.8407	59.164	107.154
CCL3	4.19163	4.0751	4.65369	8.59879	6.42075	10.6772
CCL4	1.20133	1.57657	3.21253	9.03175	7.09045	14.2899
CCL5	12.3732	6.72636	11.3247	7.79436	67.7952	61.4252
CCL7	0.593753	0.365155	0	0	0	0
CCL8	0.76813	0.546637	1.78547	2.57664	2.34504	4.00834
CCL11	0	0.312251	0.249035	0.312561	0.218778	0.150897
CCL13	19.5497	13.0316	4.55466	4.08996	7.19575	5.79962
CCL14	32.4321	20.012	24.6248	14.6478	110.854	95.6999
CCL15	0	0	0	0	0	0
CCL16	0	0.0593618	0	0	0	0.171694
CCL17	16.1575	23.5714	2.63731	15.2959	22.7675	24.1536
CCL18	13.1042	18.9635	1.95761	2.12505	6.14841	17.9126
CCL19	2.643	3.35468	0.730959	4.96349	276.058	258.609
CCL20	0.245395	0.761865	0.421471	0.211513	1.14812	1.64077
CCL21	0.9295	16.6428	1.39405	37.9362	154.991	214.286
CCL22	2.03247	2.41189	0.462472	1.30323	3.86669	3.26098
CCL23	0.475664	0.871072	0	0	0.495764	1.00169
CCL24	0	0	0	0	0	0.199392
CCL25	0.0662724	0.0474192	0.170127	0.227466	0.132593	0.0916167
CCL26	2.33241	1.99433	1.19704	2.41287	2.38796	2.1316
CCL27	0.382945	0.141319	0.169937	0	0	0
CCL28	1.10102	3.03828	1.6003	1.681	5.11393	6.70178

Gene	Patient 1		Patient 2		Patient 3	
	Center	Invasive	Center	Invasive	Center	Invasive
CCR1	1.96008	1.51109	2.17868	1.5796	6.45902	5.2881
CCR2	1.80703	1.6066	0.72301	0.612565	6.96145	6.63346
CCR3	0.251468	0.0254648	0.273719	0.12189	0.640598	0.368181
CCR4	0	0.0529927	0.0632802	0.0634108	0.703479	1.78755
CCR5	0.618147	0.593854	0.578292	0.680134	5.32627	5.01458
CCR6	0.733909	1.1529	0.714649	0.943823	2.81944	4.3377
CCR7	0.581016	0.606908	0.1402	0.351034	5.74067	8.44654
CCR8	0	0.179433	0.107159	0.179015	0.125414	0.317162
CCR9	0	0.403138	0.260573	0.561979	0.468948	0.210227
CCR10	2.31705	1.68568	2.05818	2.59106	0.922752	0.919292

Supplementary Table 3. Gene expression of *CXCLs/CCLs* and its receptor in the cancer center and invasive border by RNA sequencing. The expression values of the genes are presented as normalized FPKM (Fragments Per Kilobase of transcript per Million fragments mapped) unit and were evaluated by the cufflinks package. Raw FPKM values were evaluated by cufflinks tool and normalized to compare the expression between the cancer center and invasive border by cuffdiff tool in the package. Before evaluation of gene expression, raw RNA sequencing reads were cleaned by removing the potentially existing adapter sequence and mapped on the human reference genome (hg19) by tophat software.

Receptor	Cytokines	Receptor	Cytokines
CXCR1	CXCL6, 8	CCR1	CCL3, 4, 5, 7, 14, 15, 16, 23
CXCR2	CXCL1, 2, 3, 5, 6, 7, 8	CCR2	CCL2, 7, 8 ,12, 13
CXCR3	CXCL4, 9, 10, 11	CCR3	CCL5, 7, 11, 13, 15, 24, 26, 28
CXCR4	CXCL 12	CCR4	CCL2, 3, 5, 17, 22
CXCR5	CXCL13	CCR5	CCL3, 4, 5, 8
CXCR6	CXCL16	CCR6	CCL20
CXCR7	CXCL11, 12	CCR7	CCL19, 21
		CCR8	CCL1, 4, 17
		CCR9	CCL25
		CCR10	CCL27, 28

Supplementary Table 4. CXCLs/CCLs and their receptor.

	Lymph node (-)	Lymph node (+)
BRAFV600E PTC	39% (31/79 cases)	61% (48/79 cases)

Supplementary Table 5. Percentage of lymph node metastasis in BRAFV600E expressing PTC. Metastatic regional lymph nodes of 79 cases of BRAFV600E PTC was analyzed and presented as a table.

Genes	Sense primer 5’-3’	Antisense primer 5’-3’
**MMP1**	CTGCTTACGAATTTGCCGACAGA	GTTCTAGGGAAGCCAAAGGAGCTG
**MMP3**	GGACAAAGGATACAACAGGGACCA	GAACCGAGTCAGGTCTGTGAGTG
**MMP9**	CACTGACGTCTTCCAGTACCGAGA	CATAGGTCACGTAGCCCACTTGGT
**CXCL1**	CTTGCCTCAATCCTGCATC	CCTTCTGGTCAGTTGGATTTG
**CXCL2**	TGCAGGGAATTCACCTCAAG	TGAGACAAGCTTTCTGCCCA
**CXCL3**	CCAAACCGAAGTCATAGCCACACT	ACTTCTCTCCTGTCAGTTGGTGCT
**CXCL5**	TCTACACCAGTGGCAAGTGCT	TCCCGAACCCATTTCTTCTC
**CXCL6**	TTTGTCTGGACCCGGAAGC	CGCTGAAGACTGGGCAATTT
**CXCL10**	TGAAATTATTCCTGCAAGCCAA	CAGACATCTCTTCTCACCCTTCTTT
**CXCL12**	TGCCAGAGCCAACGTCAAG	CAGCCGGGCTACAATCTGAA
**CXCR1**	TGCATCAGTGTGGACCGTTA	TGTCATTTCCCAGGACCTCA
**CXCR2**	TGCATCAGTGTGGACCGTTA	CCGCCAGTTTGCTGTATTG
**CXCR3**	GGAGCTGCTCAGAGTAAATCAC	GCACGAGTCACTCTCGTTTTC
**CXCR4**	GCCTTATCCTGCCTGGTATTGTC	GCGAAGAAAGCCAGGATGAGGAT
**CXCR7**	GGCTATGACACGCACTGCTACA	TGGTTGTGCTGCACGAGACT
**CCL2**	CTCTGCCGCCCTTCTGTG	TGCATCTGGCTGAGCGAG
**CCL20**	GGCTATGACACGCACTGCTACA	TGGTTGTGCTGCACGAGACT
**IL-6**	GGTACATCCTCGACGGCATCT	GTGCCTCTTTGCTGCTTTCAC
**IL-8**	ATGACTTCCAAGCTGGCCGTGGCT	TCTCAGCCCTCTTCAAAAACTTCTC
**IL-1β**	CGACAGAATCTAGTTGTCC	TCATAAACACTCTCATCCACAC
**VEGF**	TGAATGCAGACCAAAGAAAGATAG	CACGTCTGCGGATCTTGTACAA
**$p16^{INK4A}$**	CCCAACGCACCGAATAGTTA	ACCAGCGTGTCCAGGAAG
**18s**	CGGCTACCACATCCAAGGAA	GCTGGAATTACCGCGGCT
**β-actin**	CCCTGGCACCCAGCAC	GCCGATCCACACGGAGTAC

Supplementary Table 6. The primers used for real-time PCR.
